# Laparoscopic Cholecystectomy in a Patient With Situs Inversus: A Case Report and Literature Review

**DOI:** 10.7759/cureus.50598

**Published:** 2023-12-15

**Authors:** Jennifer Song, Robert Fincher, Michael McCool, Andrew McCague, Paul Wisniewski

**Affiliations:** 1 Osteopathic Medicine of the Pacific, Western University of Health Sciences, Pomona, USA; 2 Trauma, Desert Regional Medical Center, Palm Springs, USA

**Keywords:** congenital abnormalities, autosomal recessive disorders, gallstones complication, conventional laparoscopic cholecystectomy, complete situs inversus

## Abstract

Situs inversus (SI) is an autosomal recessive congenital abnormality in which there is a complete mirror reversal of visceral organs. In this article, we present the case of a 26-year-old male with a past medical history of suicidal ideations, gallstones, and SI who complained of left upper quadrant pain for two weeks. After admission for acute cholecystitis, he underwent a successful laparoscopic cholecystectomy without postoperative complications. Due to the anatomical deviation characteristic of SI, it can be challenging for surgeons to accurately diagnose and perform laparoscopic cholecystectomies. Careful consideration must be given when deciding to do a laparoscopic cholecystectomy, as the placement of not only the trocars and surgical instruments but also the position of the surgeon and assistants needs to be deliberated.

## Introduction

Situs inversus (SI) is an autosomal recessive congenital abnormality characterized by a complete mirror reversal of both the abdominal and the thoracic organs [1]. The normal human body’s composition is termed situs solitus with the classic external human body demonstrating bilateral symmetry with internal asymmetry [1]. Even paired organs that we commonly think of, such as the lungs and kidneys, show some degree of asymmetry. This degree of asymmetry is especially important for surgeons as procedures can differ due to varying degrees of vascularity and the composition of the anatomy. In this article, we present a case of a 26-year-old male with a past medical history of suicidal ideations and gallstones complaining of left upper quadrant pain for two weeks.

## Case presentation

A 26-year-old male presented to the emergency department with left upper quadrant and epigastric abdominal pain for two weeks. He had been diagnosed with gallstones two years prior but never pursued surgery. His medical history was significant for SI and gallstones with a previous history of a laparoscopic appendectomy. He was admitted for management of acute cholecystitis.

**Figure 1:**
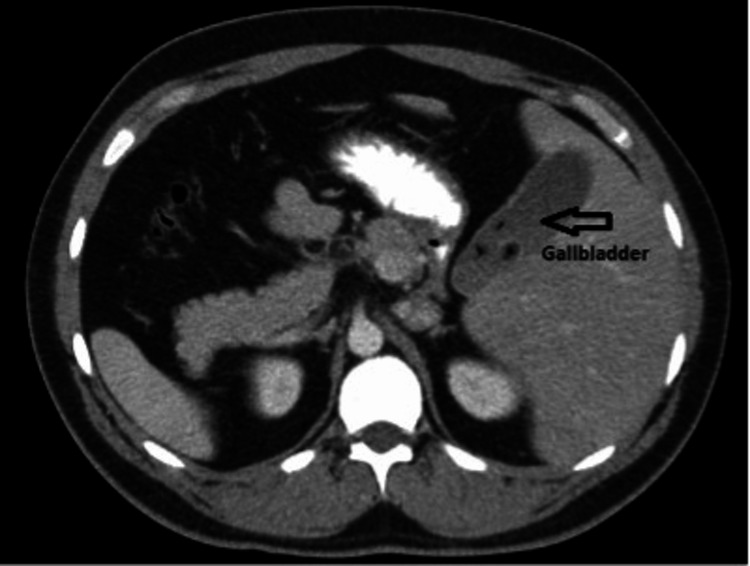
CT scan of the patient with SI. Stones in the gallbladder can be seen The surgeon was on the patient's right side and the assistant on the patient's left side

Pertinent admitting labs included a complete blood count within normal limits, electrolytes within normal limits, elevated glucose, alkaline phosphatase within normal limits, alanine transaminase in the upper limit of normal, aspartate transaminase within normal limits, lipase within normal limits, and total bilirubin in the upper limit of normal (Table 1). A CT scan and abdominal ultrasound were performed, which showed gallbladder wall thickening and gallstones suggesting cholecystitis (Figure 1).

**Table 1:** Labs at the time of presentation WBC: white blood cells, ALT: alanine transaminase, AST: aspartate transaminase

Parameters	Results	Reference range
Hemoglobin (g/dL)	15.8	Male 13.5-17.5 g/dL
WBC (cells/mcL)	10.7	4.5-11
Platelet (×10^9^)	278	150-400
Glucose (mg/dL)	116	70-110
Hematocrit	45.4%	Male 41-53%
Sodium (mEq/L)	136	136-146
Potassium (mEq/L)	3.7	3.5-5.0
Chloride (mEq/L)	98	95-105
Bicarbonate (mEq/L)	23	22-28
Total Bilirubin (mg/dL)	0.9	0.1-1.0
ALT (U/L)	40	10-40
AST (U/L)	27	12-38
Alkaline phosphatase (U/L)	81	25-100
Lipase (U/L)	39	0-160

**Figure 2:**
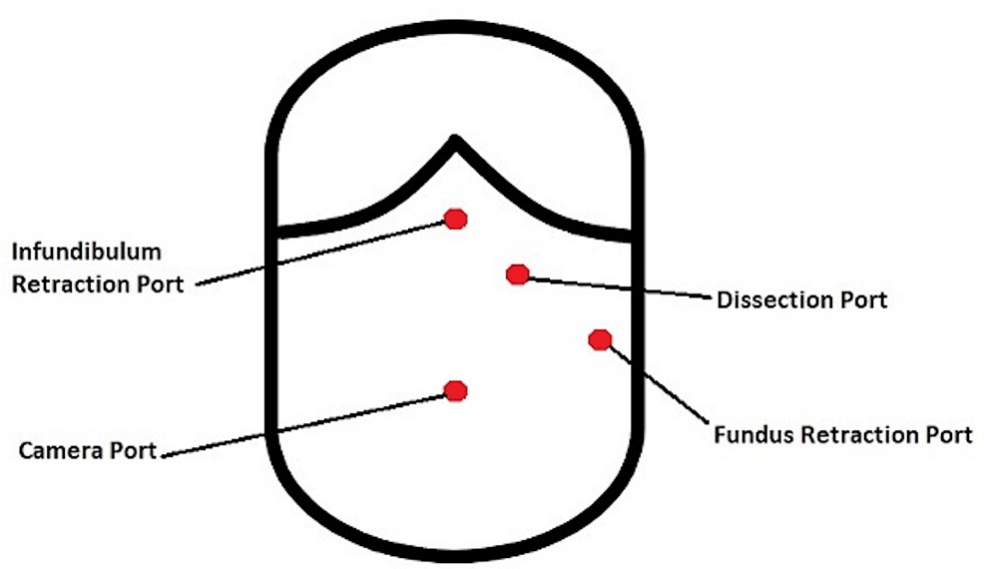
Port sites for laparoscopic cholecystectomy in the patient with SI Image credits: Andrew McCague

While admitted, he was scheduled for a laparoscopic cholecystectomy. He was taken to the operating room on the same day as his presentation. He was placed on the operating room table, intubated, and started on general anesthesia. His abdomen was prepped and draped in a sterile fashion. A small incision was made just above the umbilicus, and a Veress needle was used to insufflate the abdomen to 15 mmHg. An additional 12-mm port was placed in the epigastric region, and two 5-mm ports were placed in the left upper quadrant. The abdomen was briefly explored. There were no signs of an iatrogenic injury. The gallbladder was grasped and retracted. It had omental adhesions that were taken down. The cystic duct and cystic artery were dissected circumferentially, clipped, and ligated. The gallbladder was removed from the gallbladder fossa using an electrocautery. The gallbladder was then removed from the abdomen in an EndoCatch bag. The epigastric port site was closed with a 0-Vicryl suture using a Carter-Thomason device, and the skin was closed with a 4-0 Monocryl. The patient was extubated, transferred to a gurney, and taken to the postanesthesia care unit for recovery. Figure 2 demonstrates the port site placement. He had an uneventful postoperative recovery and was discharged home the following day.

## Discussion

SI is a rare autosomal recessive congenital abnormality characterized by a complete mirror reversal arrangement of the internal organs along the left-right axis [2]. While it is difficult to estimate the real frequency of this abnormality, a systematic review from Eitler et al. found that there was a 1:10,000 prevalence rate, with similar rates found in Adams et al. in 1937 and Lin et al. in 2000 [3].

One of the first reported cases of SI was by Aristotle who noticed this reversal in animals. In the 17th century, Fabricius and Blalock described the first case of a person with liver and spleen reversal, and Kuchenmeister in the 19th century initially recognized the condition in a living person through a thorough physical exam [3]. In the modern era, initial imaging with ultrasound or plain films is the method of choice to view and clarify this abnormality [3].

Upon literature review, the first successful case of a laparoscopic cholecystectomy was performed by Mouret in 1987, whose technique has now become the gold standard [4]. In 1991, the first successful case of laparoscopic cholecystectomy in a patient with SI was performed by Campos and Sipes [4]. Since then, 91 successful cases have been reported in the literature without significant postoperative complications [4]. Thus, SI is not a contraindication to laparoscopic cholecystectomy. However, careful consideration must be met when deciding to operate on a patient with SI due to the left-right mirror reversal of the visceral organs that can make it difficult for an accurate diagnosis and surgical approach [5]. While SI does not predispose one to increased gallbladder disease, it can lead to diagnostic confusion as most patients present with left upper quadrant abdominal pain [5]. Ultrasonography or plain film X-rays are usually the initial choice for imaging, and CT or MRI can be used to view more detailed anatomy and possible pathological findings [5].

While there is no specific standard of operating procedure for laparoscopic cholecystectomy in patients with SI, the position of the trocars and consideration of the handedness of the lead surgeon are of utmost importance. In addition, rearrangement of surgical equipment and allowing extra time to recognize the mirrored visceral organs to prevent iatrogenic injury are crucial to success in the operating room [5]. The advantage of doing laparoscopic procedures is that they can be modified and customized to a patient’s varying anatomy without significant deviation from standard procedures [4]. The most widely used technique for patients with SI is having the surgeon and camera assistant stand on the right side of the patient, while the first assistant and monitor are situated on the left [1]. Salama et al. utilized two ports placed in the epigastric and subumbilical areas and two ports placed in the left midclavicular and left anterior axillary lines [5]. Eitler et al. utilized the technique of placing an umbilical trocar for the camera and three trocars in the standard subcostal positions but on the left rather than the standard right [3]. The surgeon can then hold the infundibulum (Hartmann’s pouch) with the left hand through the subxiphoid port and use the right hand to perform the dissection through the left midclavicular port [3].

While there is a preferential bias for left-handed surgeons operating on patients with SI, utilization of good ergonomics, solid knowledge of anatomy, and rearrangement of surgical instruments are good markers of a successful operation.

## Conclusions

This case represents a rare example of surgical management of cholecystitis in a patient with SI. Accurate diagnosis of cholecystitis can be made with a thorough understanding of anatomy as well as the utilization of imaging such as ultrasound and plain films. Once a diagnosis is made and the patient elects to undergo a laparoscopic cholecystectomy, extra time must be utilized to prepare an operating room that is ergonomically efficient for the surgeon and the team. A significant deviation from the standard procedure is not necessary except for the rearrangement of tools, the placement of trocars, and the position of the surgeon and assistants, which must be carefully considered. Despite the challenging nature of such a case, SI is not a contraindication to laparoscopic cholecystectomy.
